# Supplementary material for: Structural basis for EtfD-mediated coupling of β-oxidation and the respiratory chain in mycobacteria
Source: EMBO J. 2026 Mar 17;45(8):2785–807. doi: 10.1038/s44318-026-00726-y (PMC13083937; doi:10.1038/s44318-026-00726-y)
Supplement: Supplementary file 1 — Table EV1 [file 44318_2026_726_MOESM1_ESM.docx]

**Table EV1.** Cryo-EM data collection, refinement, and validation statistics.

|  | EtfD  (EMD-70545, PDB 9OJN) | EtfD – soluble region  (EMD-70546) |
| --- | --- | --- |
| Data collection and processing | | |
| Microscope | Titan Krios G3 | |
| Detector | Falcon 4i | |
| Automation software | EPU | |
| Magnification | 120,000 | |
| Voltage (kV) | 300 | |
| Electron exposure (e^-^/Å^2^) | 70 | |
| Exposure rate (e^-^/pixel/s) | 7.7 | |
| Exposure time (s) | 3.7 | |
| Defocus range (µm) | 0.8-2.3 | |
| Pixel size (Å) | 0.64 | |
| Movies used for processing (no.) | 11,952 | |
| Symmetry imposed | C1 | |
| Initial particle images (no.) | 619,708 | |
| Final particle images (no.) | 48,053 | 151,738 |
| Map resolution (Å) |  |  |
| FSC threshold | 0.143/0.5 | 0.143/0.5 |
| Masked | 3.2/3.5 | 2.8/3.2 |
| Unmasked | 3.7/7.3 | 3.5/7.4 |
| Map resolution range (Å) | 2.9-5.0 | 2.5-3.1 |
|  |  |  |
| Refinement | |  |
| Initial model used (accession code) | AF-A0QQB0-F1-v4 |  |
| Model resolution (Å) | 3.3 |  |
| FSC threshold | 0.5 |  |
| Map sharpening *B* factor (Å^2^) | -65.6 | -98.7 |
| Model composition | |  |
| Non-hydrogen atoms | 5,601 |  |
| Protein residues | 742 |  |
| Ligands | 1 LMT  1 MQ9  1 HEM  2 SF4  1 A1CBX  1 9S8 |  |
| R.m.s deviations | |  |
| Bond lengths (Å) | 0.004 |  |
| Bond angles (°) | 0.954 |  |
| Validation | |  |
| MolProbity score | 1.22 |  |
| Clashscore | 4.40 |  |
| Rotamer outliers (%) | 0.00 |  |
| CaBLAM outliers (%) | 0.68 |  |
| EMRinger score | 4.48 |  |
| Average Q-score | 0.528 |  |
| CC (volume) | 0.78 |  |
| CC (mask) | 0.80 |  |
| Ramachandran plot |  |  |
| Favored (%) | 98.1 |  |
| Allowed (%) | 1.90 |  |
| Outliers (%) | 0.00 |  |
