# Supplementary material for: Structural basis for EtfD-mediated coupling of β-oxidation and the respiratory chain in mycobacteria
Source: EMBO J. 2026 Mar 17;45(8):2785–807. doi: 10.1038/s44318-026-00726-y (PMC13083937; doi:10.1038/s44318-026-00726-y)
Supplement: Supplementary file 3 — Movie EV1 [file 44318_2026_726_MOESM3_ESM.zip › Movie EV1_Legend.docx]

**Movie EV1.** Cryo-EM map density and atomic models of the iron-sulfur clusters in EtfD.
