# Supplementary material for: Structural basis for EtfD-mediated coupling of β-oxidation and the respiratory chain in mycobacteria
Source: EMBO J. 2026 Mar 17;45(8):2785–807. doi: 10.1038/s44318-026-00726-y (PMC13083937; doi:10.1038/s44318-026-00726-y)
Supplement: Supplementary file 6 — Expanded View Figures [file 44318_2026_726_MOESM6_ESM.pdf]

Expanded View Figures

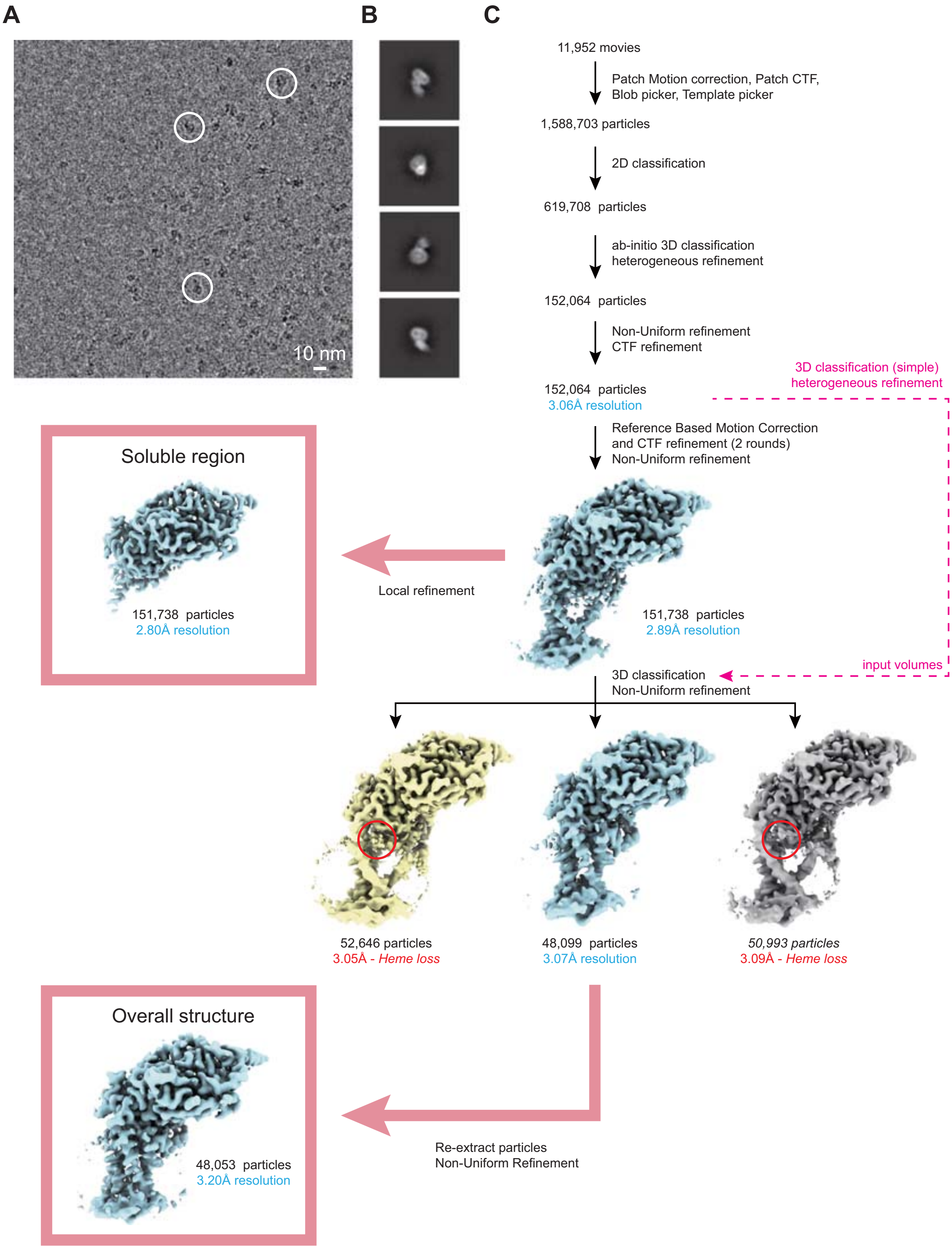

Figure EV1. Cryo-EM workflow.

(**A**) Representative micrograph. Example particle images are circled. (**B**) 2D class averages of EtfD. (**C**) Simplified cryo-EM workflow. Red circles indicate the region in EtfD where the heme was lost.

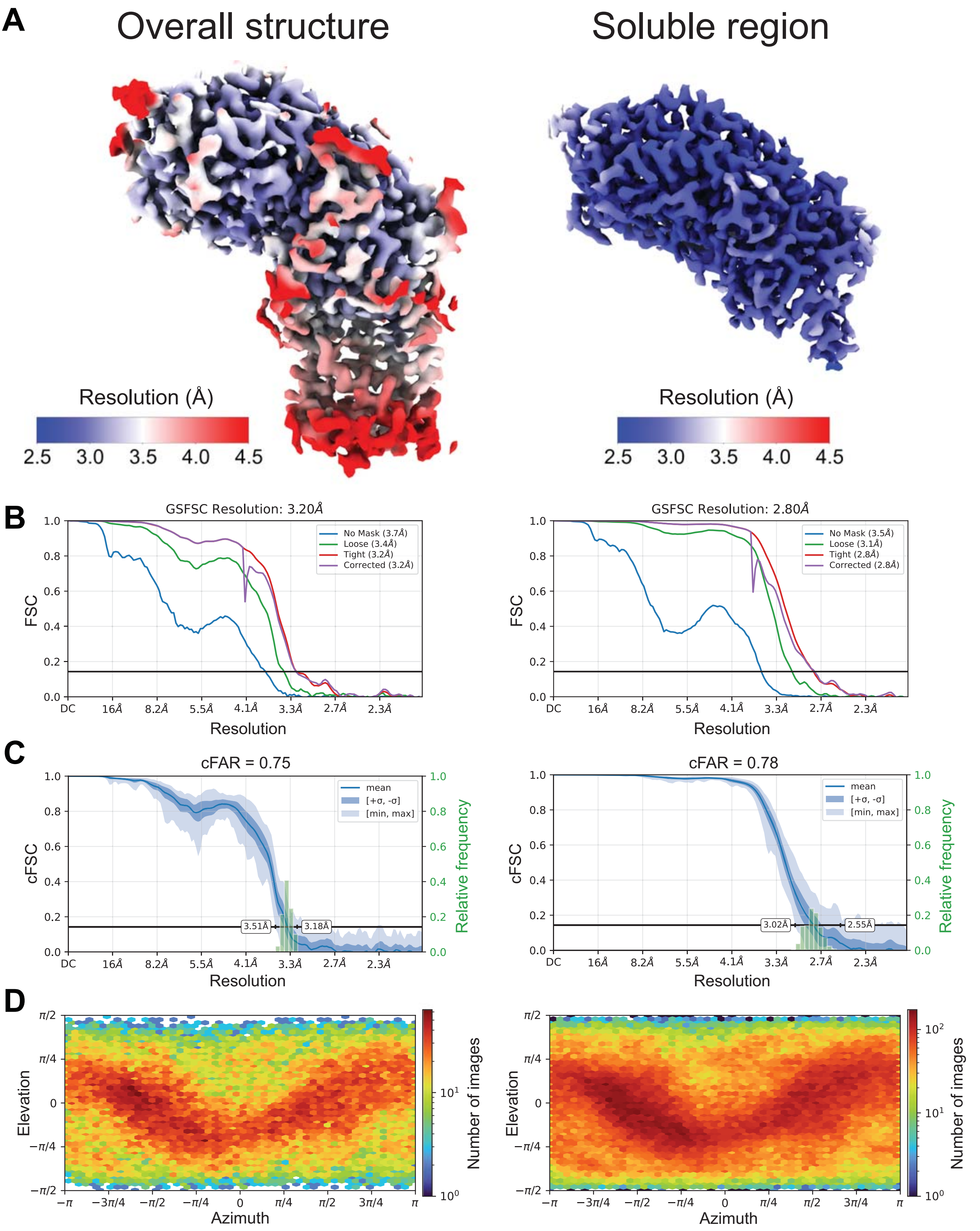
A
Overall structure
Soluble region
Resolution (Å)
2.5 3.0 3.5 4.0 4.5
Resolution (Å)
2.5 3.0 3.5 4.0 4.5
B
GSFSC Resolution: 3.20Å
No Mask (3.7Å)
Loose (3.4Å)
Tight (3.2Å)
Corrected (3.2Å)
FSC
Resolution
GSFSC Resolution: 2.80Å
No Mask (3.5Å)
Loose (3.1Å)
Tight (2.8Å)
Corrected (2.8Å)
FSC
Resolution
DC 16Å 8.2Å 5.5Å 4.1Å 3.3Å 2.7Å 2.3Å
C
cFAR = 0.75
mean
[+σ, -σ]
[min, max]
3.51Å
3.18Å
cFSC
Relative frequency
Resolution
cFAR = 0.78
3.02Å
2.55Å
D
Elevation
Azimuth
Number of images
−π −3π/4 −π/2 −π/4 0 π/4 π/2 3π/4 π
π/2 π/4 0 −π/4 −π/2

 © The Author(s)

Figure EV2. Cryo-EM map validation.

(**A**) Local resolution map for the full EtfD structure (*left*) and the locally refined soluble region of EtfD (*right*). (**B**) Fourier Shell Correlation (FSC) curves obtained after gold-standard refinement. (**C**) Conical FSC (cFSC) plots and their associated cFSC area ratio (cFAR) scores. (**D**) Particle orientation distribution plots.

© The Author(s)

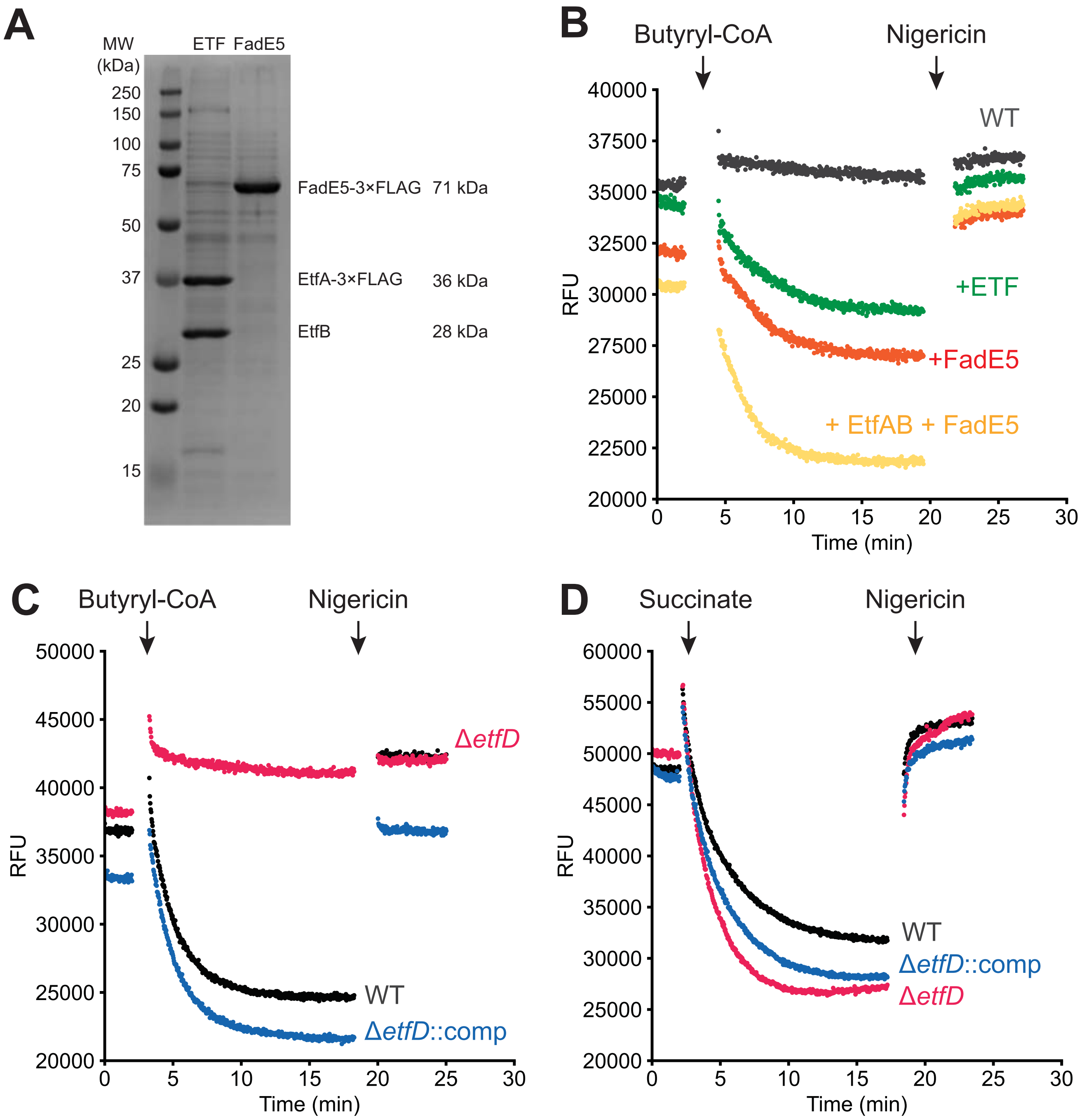

Figure EV3. Assay replicates.

(**A**) SDS-PAGE gels of purified ETF and FadE5. (**B**) Butyryl-CoA-driven proton pumping activity in IMVs prepared from wild-type *M. smegmatis* (GMC_MSM1) supplemented with either buffer (*black*), ETF (*green*), FadE5 (*orange*), or ETF and FadE5 (*yellow*). (**C**) Butyryl-CoA-driven proton pumping activity in the presence of ETF and FadE5 for IMVs prepared from *M. smegmatis* that is either wild-type (*black*), Δ*etfD* (*red*) (GMC_MSM9), or Δ*etfD* complemented with *M. tuberculosis* EtfD (GMC_MSM10). (**D**) Succinate-driven proton pumping activity in IMVs prepared from *M. smegmatis* that is either wild-type (*black*), Δ*etfD* (*red*), or Δ*etfD* complemented with *M. tuberculosis* EtfD (*blue*).

 © The Author(s)

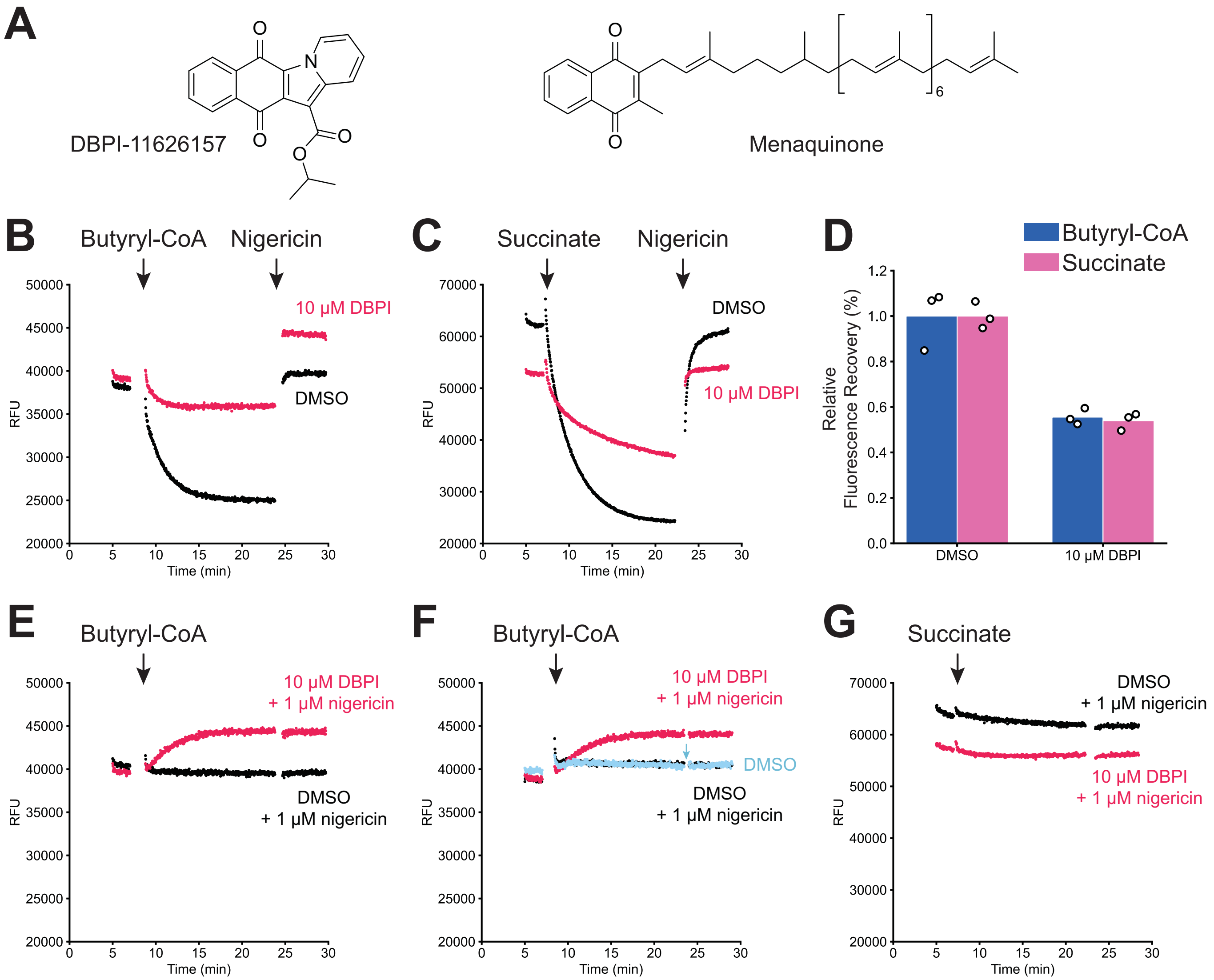

Figure EV4. Effect of DBPI-11626157 on butyryl-CoA-driven and succinate-driven IMV acidification.

(**A**) Chemical structure of DBPI-11626157 (*left*) and menaquinone (*right*). (**B**) Butyryl-CoA-driven acidification of Δ*etfD*::*rv0338c* IMVs in the presence of 2 µM ETF and 1 µM FadE5, with 10 µM DBPI-11626157 (*red*) or DMSO (*black*). Data is representative of three replicates. (**C**) Succinate-driven acidification of Δ*etfD*::*rv0338c* IMVs with 10 µM DBPI-11626157 (*red*) or DMSO (*black*). Data is representative of three replicates. (**D**) Quantification of butyryl-CoA-driven and succinate-driven IMV acidification assays. (**E**) Addition of butyryl-CoA to Δ*etfD*::*rv0338c* IMVs in the presence of 1 µM nigericin, 2 µM ETF, and 1 µM FadE5, with 10 µM DBPI-11626157 (*red*) or DMSO (*black*). Data is representative of three replicates. (**F**) Addition of butyryl-CoA to Δ*etfD*::*rv0338c* IMVs without FadE5 or ETF with 10 µM DBPI-11626157 and 1 µM nigericin (*red*), DMSO and 1 µM nigericin (*black*), or DMSO alone (*blue*). Nigericin (1 µM) was added to the DMSO-only well during the assay (*blue arrow*). Data is representative of two replicates. (**G**) Addition of succinate to Δ*etfD*::*rv0338c* IMVs in the presence of 1 µM nigericin with 10 µM DBPI-11626157 (*red*) or 1 µM nigericin and DMSO (*black*). Data is representative of three replicates.

© The Author(s)

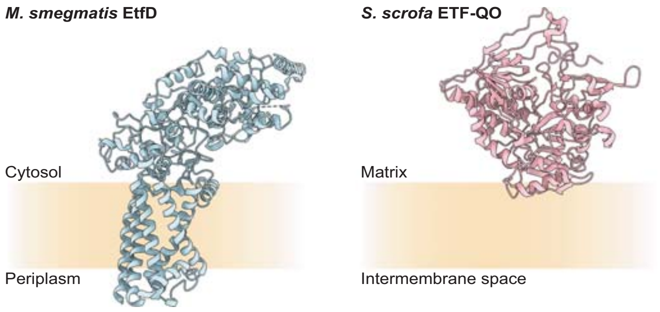

Figure EV5. Structure of mycobacterial and mammalian ETF dehydrogenases.

Comparison of EtfD from *M. smegmatis* with ETF-QO oxidoreductase from *Sus scrofa* (PDB: 2GMH).

 © The Author(s)

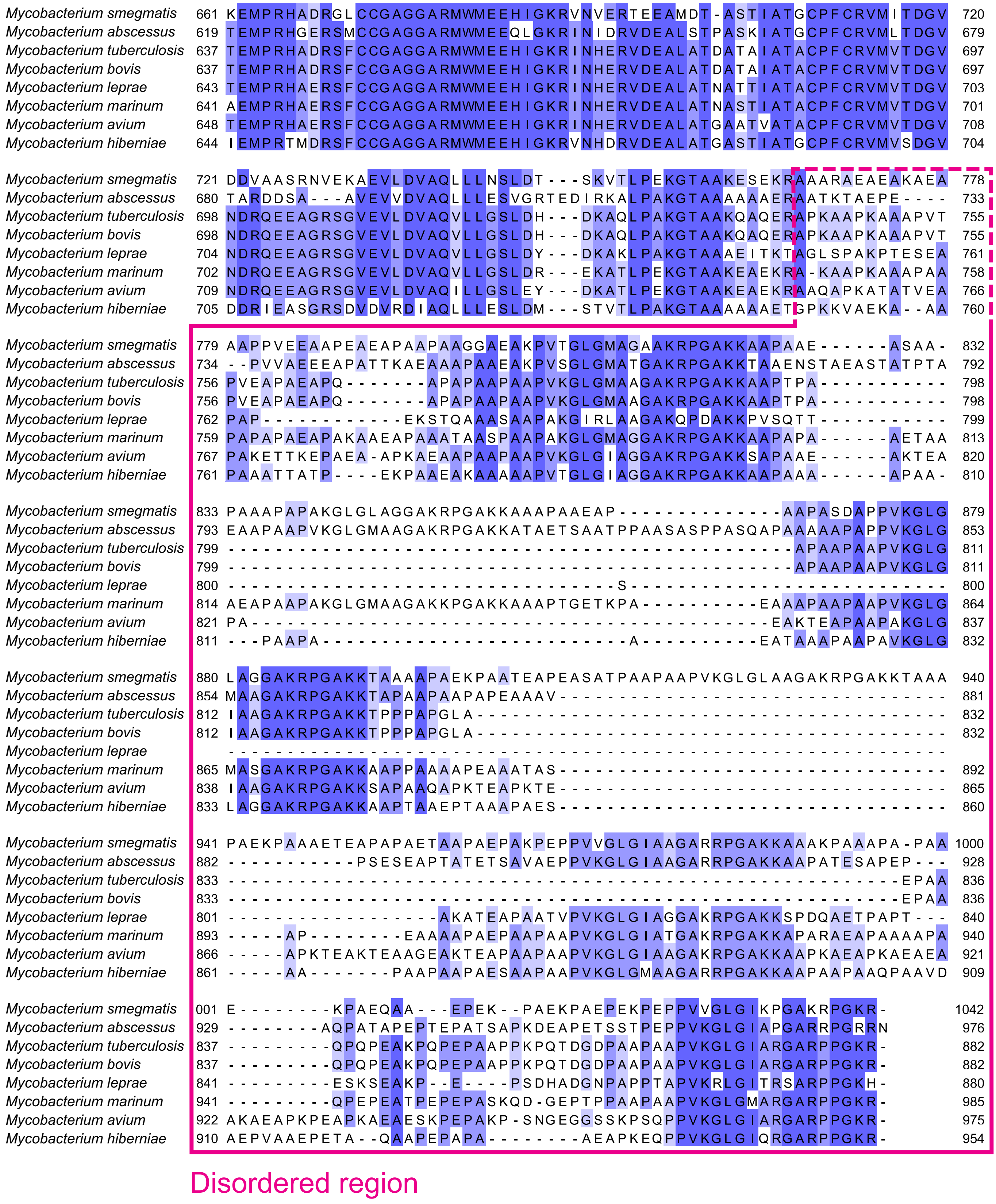

Figure EV6. Sequence alignment of the disordered region of mycobacterial EtfD homologs.

Sequences are colored by conservation. The disordered region is indicated by a pink box. The start of the disordered region differs between species (dashed line).

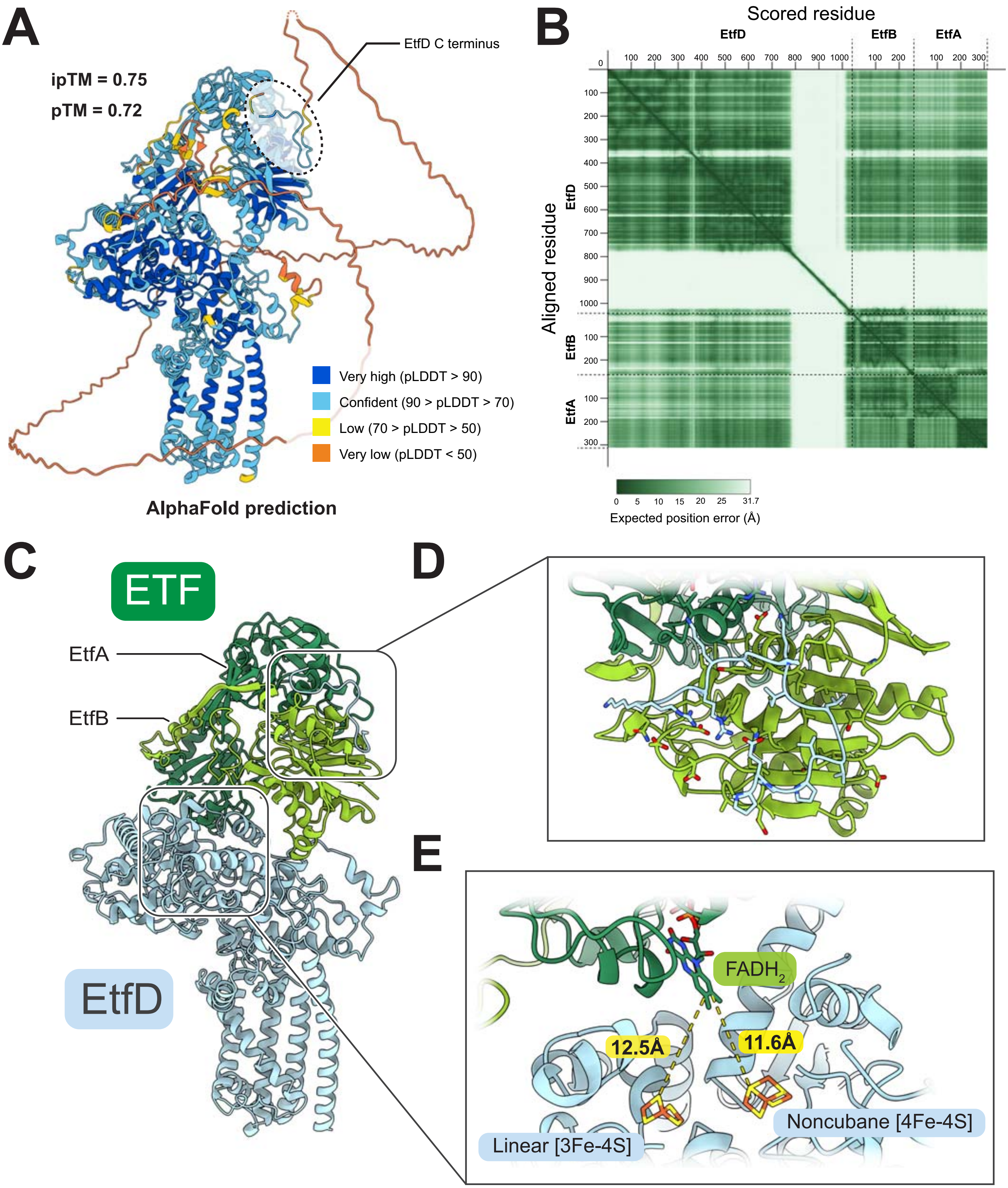

Figure EV7. AlphaFold prediction of the ETF:EtfD complex.

(**A**) AlphaFold 3 prediction of the ETF-EtfD complex structure, colored by pLDDT score. (**B**) Predicted aligned error plot (Elfmann and Stülke, 2023). (**C**) Overview of the interaction between ETF (*green*) and EtfD (*blue*). The ~30 kDa disordered region of EtfD is not shown for clarity. (**D**) Close-up view of the interaction predicted by AlphaFold 3 between the disordered region of EtfD and ETF. (**E**) Location of the docked FAD cofactor of ETF from PDB 1EFV, relative to cluster D1 and D2 of EtfD.

© The Author(s)

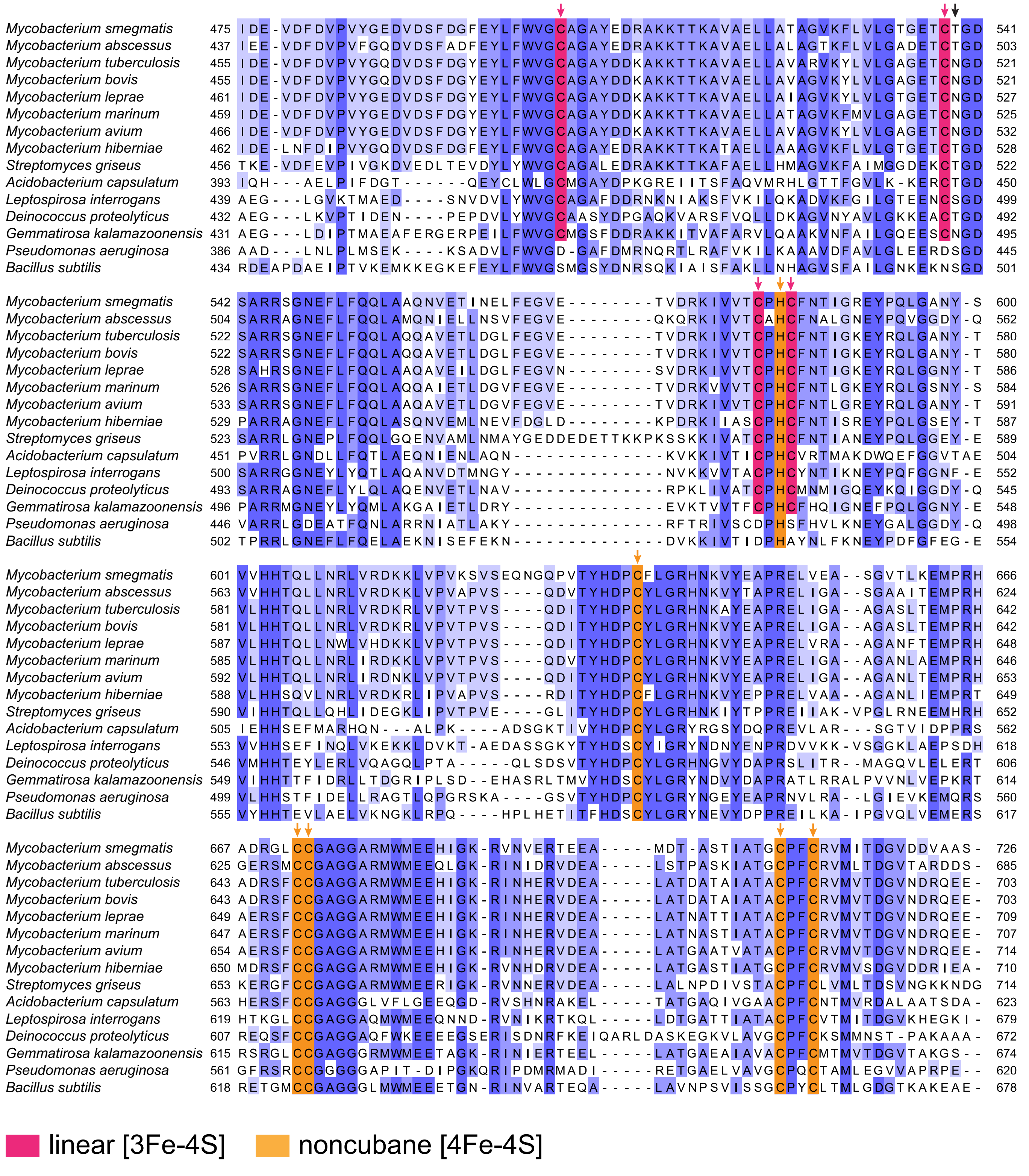

Figure EV8. Sequence alignment of the CCG domains of selected EtfD homologs.

Coordinating residues are shown with red (linear [3Fe-4S] cluster) and orange (noncubane [4Fe-4S] cluster) arrows. The black arrow shows the position of the supernumerary cysteine in Hdr. Sequences are colored by conservation.
